# It’s not always endocarditis: splinter hemorrhages in ANCA-associated vasculitis

**DOI:** 10.1007/s00508-026-02734-8

**Published:** 2026-03-19

**Authors:** Werner Ribitsch, Alexandra Radler

**Affiliations:** Division of Nephrology, Department of Internal Medicine, Medical University of Graz, Auenbruggerplatz 17, 8036 Graz, Austria

A 59-year-old woman was referred to the emergency department due to dyspnea with respiratory insufficiency and productive cough. Approximately 1 month prior to admission she contracted an infection from severe acute respiratory syndrome coronavirus 2 (SARS-CoV-2) with subsequent occurrence of a painful, swollen and reddened ankle. A postinfection arthritis was suspected by an external hospital and she was started on a high-dose methylprednisolone treatment.

**Fig. 1:**
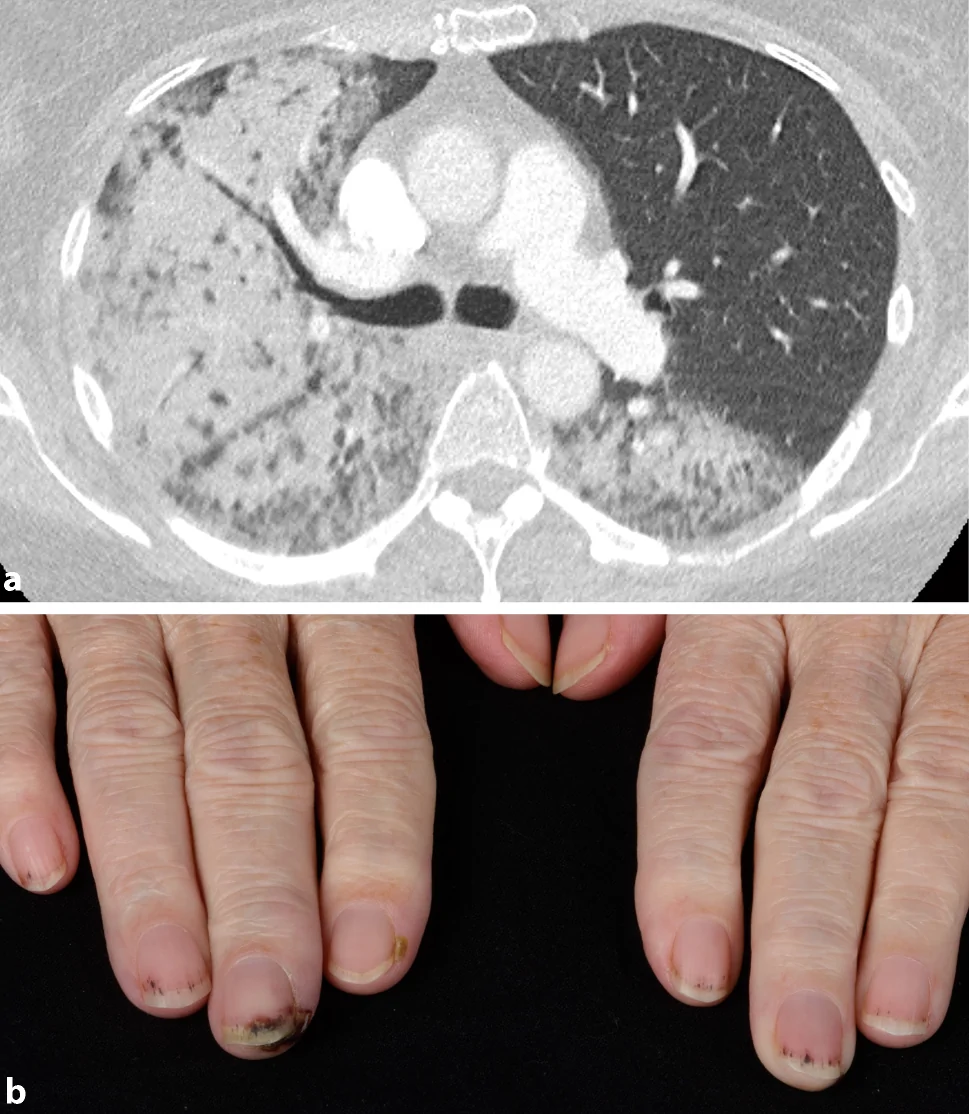
**a** Contrast-enhanced chest CT in axial lung window showing diffuse involvement of the right lung and the superior left lower lobe with ground-glass opacities. **b** Splinter hemorrhages with scattered necroses on the fingertips.

At our clinic, physical examination was remarkable for cyanosis of the fingertips and ankle edema. Laboratory tests revealed a significant decrease in hemoglobin to 7.6 g/dL, a substantially elevated C‑reactive protein (CRP) level of 312 mg/L and a nephritic sediment in the urinalysis. A chest computed tomography (CT) scan showed bilateral pulmonary infiltrates (Fig. 1a). Positive antineutrophil cytoplasmic antibodies (c-ANCA) staining by indirect immunofluorescence and PR3-ANCA detection by enzyme-linked immunosorbent assay, together with a renal biopsy showing pauci-immune rapidly progressive glomerulonephritis, provided the diagnosis of granulomatosis with polyangiitis. Immunosuppressive induction therapy with high dose prednisone, cyclophosphamide, rituximab and plasmapheresis was started. During the first few days of treatment, the patient developed splinter hemorrhages with scattered necroses of the fingertips (Fig. 1b).

Endocarditis as the cause of these splinter hemorrhages was excluded by transesophageal echocardiography and a source of thromboembolic spread was ruled out by magnetic resonance imaging. We therefore defined these lesions as a manifestation of granulomatosis with polyangiitis. After completing induction therapy, the respiratory insufficiency and kidney function significantly improved and the splinter hemorrhages started to resolve. The patinet was discharged after a stay of 26 days in our hospital.

